# Mechanism of T7 Primase Selecting Active Priming Sites Among Genome

**DOI:** 10.3390/biom16010078

**Published:** 2026-01-03

**Authors:** Zhiming Zhang, Jiang Chen, Wenyue Liu, Yu Wang, Haoyang Cai, Ganggang Wang

**Affiliations:** 1 Agricultural Microbial Agents Key Laboratory of Sichuan Province, Chengdu Institute of Biology, Chinese Academy of Sciences, Chengdu 610213, China; zhangzm@cib.ac.cn (Z.Z.); chenjiang2019@163.com (J.C.); wenyueliu@whu.edu.cn (W.L.); 2 University of Chinese Academy of Sciences, Beijing 100049, China; 3 College of Life Sciences, Sichuan University, Chengdu 610064, China; wangyu@stu.swun.edu.cn (Y.W.); haoyang.cai@scu.edu.cn (H.C.)

**Keywords:** primase, DNA replication, specific recognition site, T7 phage, flanking sequence

## Abstract

In bacteriophage T7, the primase synthesizes primer at a specific site, 5′-(G/T)_2_GTC-3′. However, the pentanucleotide alone cannot define the activity of the primase. In this study, we demonstrated that the 10-nt sequence flanking the 3′ end of pentanucleotide sites made considerable contributions to the interactions between T7 primase and single-strand DNA (ssDNA). Approximately 26 template sequences with multiple features were screened out from the T7 genome, which exhibited strong binding affinity to T7 primase and high priming activity, thus supporting genome replication. Notably, a dinucleotide in the 3′ flank of the pentanucleotide site was found to be instrumental in T7 primase binding to ssDNA, which might be recognized by the zinc-binding domain of T7 primase. As a result, a multiple-site recognition model for T7 primase to select priming sites was proposed. These results shed light on how T7 primase selects priming sites, a process that may be shared by its bacterial counterparts. Furthermore, our study provides novel methodologies for investigating the interactions between prokaryotic primases and their ssDNA templates, thereby laying the groundwork for the development of novel inhibitors.

## 1. Introduction

In DNA replication, DnaG primases synthesize short RNA primers, which are extended by DNA polymerases to form Okazaki fragments [1,2,3,4,5,6]. DnaG primases are composed of a zinc-binding domain (ZBD), an RNA polymerase domain (RPD), and a helicase-binding domain (HBD), which are primarily found in bacteria and bacteriophages [7]. For priming, DnaG primase selectively binds to a specific recognition site (SRS, usually a trinucleotide) and synthesizes the primers starting from the central base of the trinucleotide, for instance, 5′-CTG-3′ for *Escherichia coli* DnaG, 5′-CCC-3′ for *Aquifex aeolicus* DnaG, and 5′-CTA-3′ for *Bacillus subtilis* DnaG, etc. [8,9,10]. However, not all SRSs are used for priming. Kusakabe et al. reported that neither the initiation of primer synthesis nor the length of the primer was random [11]. In *E. coli*, only 3% of all 5′-CTG-3′ sites in its genome are used for priming [12]. Furthermore, Lee et al. reported the size of Okazaki fragment was not affected by the number of SRSs on the template [13]. Afek et al. demonstrated that sites with specific features exhibit stronger binding affinity to primase and produce longer primers [14]. Taken together, these results suggest that DnaG primase may select initiation sites from numerous candidate sites, rather than priming stochastically. So far, it is still elusive how the DnaG primase selects active priming site in the genome.

In bacteriophage T7, the bifunctional gp4 protein possesses primase activity within its N-terminal region (hereinafter referred to as T7 primase), a functional analog of bacterial DnaG. In T7 primase, the RPD and ZBD are linked by a flexible loop [15,16]. T7 primase initiates primer synthesis specifically at 5′-GTC-3′, the only SRS for T7 primase. This trinucleotide occurs with 504 copies across the lagging strand region of T7 genome. However, during the replication of ~37,000 bp T7 genome, the average length of Okazaki fragments is about 3000 bp, ranging from ~1000 bp to ~6000 bp [13,17,18]. This means that about 6–37 5′-GTC-3′ sites in the genome are active for priming. Only the trinucleotide 5′-GTC-3′ is insufficient to determine the DNA binding and priming specificity.

**Figure 1:**
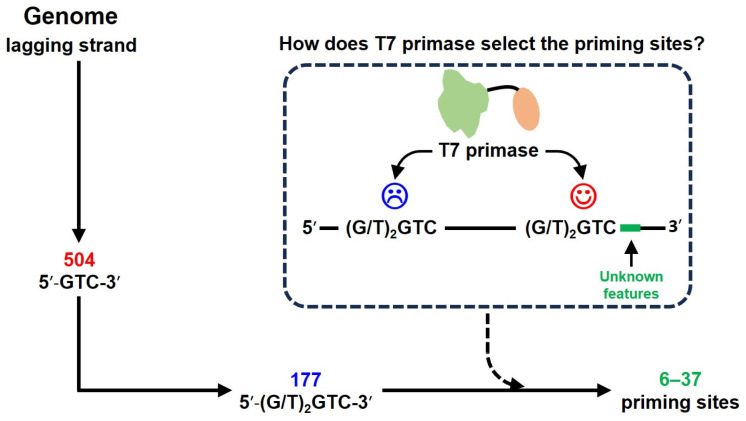
How does the T7 primase select the priming sites from pentanucleotide candidates?

By sequencing the priming products, previous studies reveal that the primers synthesized by T7 primase are 5′-ACCC-3′, 5′-ACCA-3′, 5′-ACAC-3′, and 5′-ACAA-3′ [19,20], suggesting that the specific site 5′-GTC-3′ could be expanded to pentanucleotide 5′-(G/T)_2_GTC-3′. A total of 177 pentanucleotide sites are identified in the lagging strand region of T7 genome. Notably, it is reported that the additional sequence flanking the 5′ end of pentanucleotide 5′-(G/T)_2_GTC-3′ has no effect on the T7 primase–ssDNA interactions [21]. In addition, based on a high-throughput primase profiling analysis, Afek et al. identified that the G/T-rich flanks of 5′-GTC-3′ could increase T7 primase–DNA binding affinity as well as the length of newly formed RNA primers [12,14], consistent with previous reports on T7 primase that two nucleotides flanking the 3′-end of 5′-GTC-3′ are required for tight DNA binding and rapid primer synthesis [21]. The studies on DnaG primase of *Mycobacterium tuberculosis* also shows that seven nucleotides flanking the 3′-end of the 5′-GC(G/C)-3′ site significantly influenced binding affinity [22]. Despite the above progress, the features adopted by T7 primase to select active priming sites from 177 pentanucleotide sites are still unclear (Figure 1).

In this study, based on a Sequence Iterative Optimization (SIO) strategy, we identified the features in the 3′ flanks of 5′-(G/T)_2_GTC-3′ that contribute to T7 primase–ssDNA binding specificity. Then, based on features of ssDNA containing pentanucleotide sites, the potential priming sites recognized by T7 primase in the T7 genome were screened out. Of note, the 9–10th sites in the 3′ flank of 5′-(G/T)_2_GTC-3′ might play a crucial role in the interactions between T7 primase and the ssDNA template, which might be recognized by the ZBD. Combined with the data from protein–ssDNA docking, a model for active priming sites selection by T7 primase was proposed. These results shed light on the mechanism for T7 primase when selecting active priming sites from the genome. Given that bacterial primases may share a similar recognition mechanism, this study paved the way for expanding methodologies to other bacterial DnaG primases in studying primase–ssDNA template interactions.

## 2. Materials and Methods

### 2.1. Protein Expression and Purification

Plasmid pET-28a containing T7 primase gene (813 bp, residue 1–271 of gene 4 protein) was transformed into *E. coli* BL21(DE3). The transformed cells were cultured in Luria–Bertani broth (LB) (Sangon Biotech, Shanghai, China) containing 80 μg/mL Kanamycin. At OD_600_ of ~0.6, Isopropyl β-D-1-thiogalactopyranoside (BBI life sciences corporation, Shanghai, China) was added with a final concentration of 0.2 mM and incubated at 180 rpm, 16 °C overnight. The induced cells were collected by centrifugation, then the cell pellet was resuspended in the lysis buffer [50 mM HEPES (Beyotime Biotechnology, Shanghai, China), pH 7.5, 500 mM NaCl, 10 mM MgCl_2_, 1 mM ATP, 20 mM imidazole] and lysed by sonication. The lysate was centrifuged at 7000 rpm, 4 °C for 30 min and the supernatant was then loaded onto a column of Ni-NTA. The column was washed with lysis buffer and then washed with eluent buffer [50 mM HEPES, pH 7.5, 500 mM NaCl, 10 mM MgCl_2_, 1 mM ATP, 150 mM imidazole]. The eluate was collected and diluted by dilution buffer [50 mM HEPES, pH 7.5, 10 mM MgCl_2_, 1 mM ATP, 2 mM DTT]. After that, the sample was sequentially purified by ion-exchange (Q Sepharose column, GE Healthcare) and gel filtration chromatography (Superdex 75 gel filtration column, GE Healthcare). The purified T7 primase was analyzed by SDS-PAGE with >95% purity and concentrated to >10 mg/mL for storage at −80 °C.

### 2.2. Binding Affinity Evaluation by Agarose Gel Electrophoretic Mobility Shift Assay (EMSA)

The binding affinity between ssDNA and T7 primase was evaluated by the label-free EMSA [22,23,24,25], which was widely used to study interactions between protein and nucleic acid, and the reliability of EMSA was validated by Isothermal Titration Calorimetry and Surface Plasmon Resonance [23]. In brief, the mixtures containing ssDNA and T7 primase were applied for gel analysis. ssDNA bound to protein are unable to be stained by nucleic acid dyes due to steric hindrance. Thus, band intensities only represent concentration of free ssDNA, with a more intense band indicating a higher concentration of free ssDNA and, consequently, a weaker binding affinity to T7 primase.

In this research, all ssDNA were synthesized by Sangon Biotech (Shanghai, China) Co., Ltd. The ssDNA was dissolved in ddH_2_O and quantified using Nanodrop UV spectrophotometer (Thermo Fisher Scientific, Waltham, MA, USA). Afterwards, 5 μM ssDNA and specific concentration of T7 primase were mixed in 25 mM HEPES, pH 7.5, 10 mM DTT, 10 mM MgCl_2_, and 2 mM ATP. It should be noted that either ATP or CTP could increase the affinity of the primase for its template [21], while ATP together with CTP could significantly enhance binding affinity between T7 primase and a specific template [16]. However, ATP and CTP were also the only two components required for priming. Here, we aimed to obtain a stable protein–ssDNA complex for affinity evaluation, namely, allowed binding but not priming. Thus, only ATP was used for binding affinity evaluation. The samples were incubated at 30 °C for 30 min to reach a dynamic equilibrium state and then subsequently loaded onto 2% agarose gel containing 1× UltraGelRed Stain (Nanjing Vazyme Biotech. Co., Nanjing, China). The samples were resolved by running gels in 2× TB buffer at 20 V/cm for 10 min. Sample without T7 primase was set as blank. After that, the gels were imaged in Gel Doc^TM^XR^+^ system (Bio-Rad, Hercules, CA, USA), and the relative integrated density (RID) of the free ssDNA band was measured with ImageJ 1.53t software, which was then applied to calculate the proportion of free ssDNA.

For acquisition of the apparent dissociation constant (Kd, indicating the protein concentration at 50% of free ssDNA), selected ssDNAs were incubated with various concentrations of T7 primase (0 to 150 µM), and the proportions of free ssDNA were calculated. Values for dissociation constant Kd were calculated by nonlinear fitting of the T7 primase concentrations against the intensities of free ssDNA bands.

### 2.3. Primer Synthesis Activity Analysis

The primer synthesis activity analysis was conducted by the primase-pyrophosphatase–malachite green method with modifications [26]. In brief, 125 μM T7 primase and 5 μM ssDNA were diluted with primer synthesis buffer (25 mM HEPES, pH 7.5, 150 mM KGlu, 10 mM DTT), then MgCl_2_, NTPs and pyrophosphatase at final concentrations of 10 mM, 50 μM, and 0.6 U/mL, respectively, were added and mixed. The mixture was incubated at 25 °C for 30 min followed by boiling for 1 min to denature T7 primase and pyrophosphatase. Denatured protein was removed by centrifugation at 12,000 rpm, 4 °C for 5 min. The supernatant was added to malachite green solution and incubated at 37 °C for 15 min. The absorbance at 650 nm was measured using a microplate reader. Concentration gradients of NaH_2_PO_4_ (0–600 μM) were used to establish standard curve, which was then applied to calculate amount of the newly generated Pi.

### 2.4. Sequence Iterative Optimization (SIO) Strategy

Before sequence optimization, three 28-nt ssDNA templates were extracted from regions near the origin of replication (oriC) of the T7 phage genome. As for features, these sequences are composed of 8-nt 5′ flanks, the central pentanucleotide 5′-(G/T)_2_GTC-3′, and a 15-nt 3′ flanks. The binding affinities of these ssDNAs to T7 primase were evaluated by EMSA. As a result, the ssDNA with moderate binding affinity to T7 primase was chosen as the initial one for iterative optimization.

**Figure 2:**
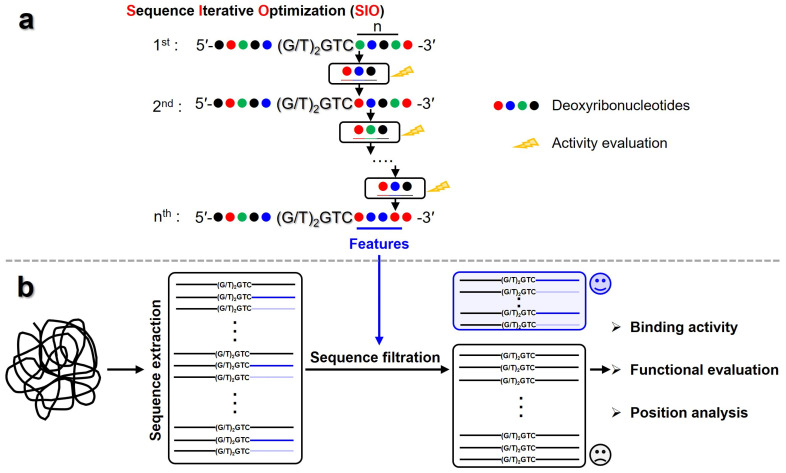
Workflow of Sequence Iterative Optimization (SIO) and Genome Screening Strategy. (**a**) SIO sketch map. The optimization was started from the first site in 3′ flanks of 5′-(G/T)_2_GTC-3′ by modifying the target deoxyribonucleotide to the other three bases, then the modification was iteratively conducted on the subsequential sites until modifications at a given site no longer produced a significant change in binding affinity. (**b**) Genome screening for potential initiation sites. Sequences containing 5′-(G/T)_2_GTC-3′ were firstly extracted from T7 phage genome, which were then filtrated based on the features obtained from the iterative optimization process. The candidate sequences were then evaluated by activity evaluation and position analysis.

The ssDNA of 5′-CCTTCAACTGGTCATACATATGGTTCAA-3′ was used for optimization. The iterative optimization was conducted on the 3′ flanks of 5′-TGGTC-3′, as illustrated in Figure 2a. For the first-round optimization, the adenine nucleoside (A) in the first site adjacent to the 5′-TGGTC-3′ was modified to T, C, or G, respectively, and the effect of the modifications on the binding affinity of ssDNA to T7 primase was evaluated by EMSA. Among them, the nucleoside favoring binding affinity of ssDNA to T7 primase was then applied for the second-round optimization and the like. The iterative optimization process was terminated when modifications at a given site no longer produced appreciable changes in ssDNA binding affinity. In total, 15 sites on the 3′ flanks of 5′-TGGTC-3′ were optimized.

### 2.5. Screening of Potential Priming Sites in the T7 Phage Genome

The genome of the T7 phage was downloaded from NCBI. All sequences containing pentanucleotide 5′-(G/T)_2_GTC-3′ were extracted. Then, the ssDNAs with specific features in the 3′ flank of the pentanucleotide site were screened out, whose binding affinity and primer synthesis activity were evaluated as described above (Figure 2b). In addition, the precise locations of the screened ssDNA were mapped onto the T7 genome, and the distance between two adjacent pentanucleotide sites were calculated by SnapGene 4.2.4. The features of the mapped ssDNA were analyzed by WebLogo 3.

### 2.6. Protein–ssDNA Docking and Interaction Analysis

The RPD and ZBD in the T7 primase crystal structure (PDB: 1NUI) [27] were used for docking. The structure of RPD in complex with ATP, CTP, and Mg^2+^ was intended to be constructed for docking. However, since CTP cannot currently be selected as a ligand for complex structure prediction in AlphaFold 3, we first predicted a complex structure comprising RPD, an RNA fragment (5′-CCCC-3′), ATP, and Mg^2+^ using AlphaFold 3 [28]. After alignment with the crystal structure of T7 primase, the cytosine ribonucleotide of RNA (5′-CCCC-3′) near the ATP among the predicted structure with the highest pLDDT score was extracted. As a result, CMP, instead of CTP, was introduced into the structure of RPD in complex with ATP and Mg^2+^, which was then applied for docking with ssDNA (5′-GGGTC-3′). The ZBD (with Zn^2+^) in T7 primase crystal structure was used for docking with ssDNA oligo 5′-TA-3′/5′-CC-3′. Docking experiments were performed using HDOCK service without explicit restraints.

Previous studies suggested that a large positive-charged patch was usually considered as the binding interface [29,30], which was defined as a selection criterion for docking. For each docking, a target structure was selected from top 100 results. The structures of complexes were visualized and analyzed with PyMOL 3.0.3 and Protein–Ligand Interaction Profiler.

### 2.7. Site-Directed Mutagenesis

Site-directed mutagenesis was performed by the QuickChange mutagenesis method [31]. Mutations were confirmed by DNA sequencing. The mutants were expressed and purified in the procedures described above.

### 2.8. Data Analysis

Protein structures were drawn using PyMOL 3.0.3. Graphing and statistical analysis were performed in GraphPad Prism v5.01. Significant differences were evaluated by one-way analysis of variance and the Tukey test, with at least three replicates. Data are expressed as the mean ± standard error of the mean (SEM).

## 3. Results

### 3.1. 3′ Flanks of 5′-(G/T)_2_GTC-3′ Contributes to the Binding Affinities of T7 Primase to ssDNA

Three ssDNA templates mentioned in Section 2.4 were extracted from the region around the oriC of the T7 phage genome, which were named as sD1, sD2, and sD3 for sequence optimization. The binding affinities of ssDNA to T7 primase were measured by EMSA, accompanied with a negative control ssDNA (sD4) containing non-canonical site 5′-CGGTC-3′ (Table S1). As shown in Figure S1, the ssDNA of sD1, sD2, and sD3 exhibited different binding affinities to T7 primase, while sD4 showed no detectable binding affinity. Among them, sD3 exhibited higher binding affinity to T7 primase than sD1 and sD2. Hence, sD3 was chosen for SIO.

**Figure 3:**
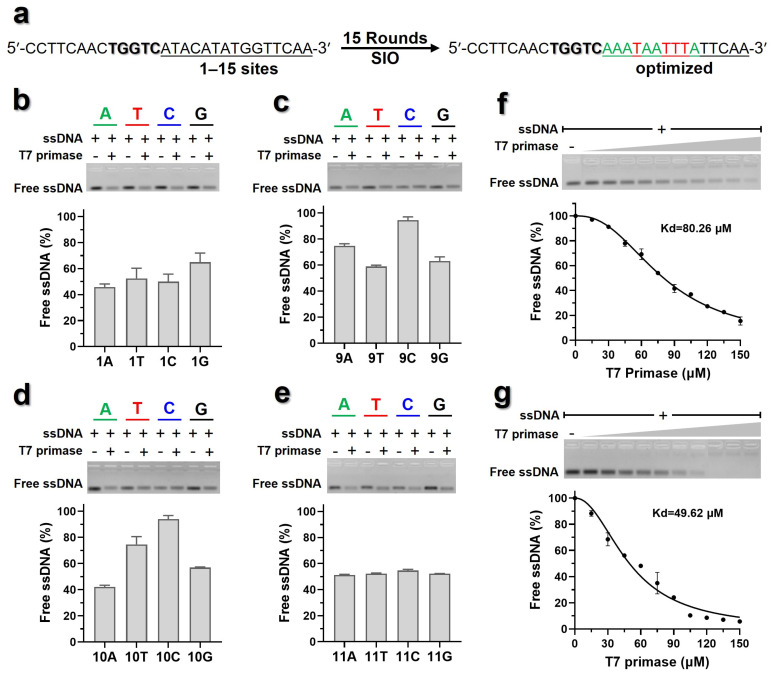
SIO on ssDNA sD3. (**a**) Schematic of the iterative optimization workflow. The origin sequence (left) was iteratively optimized to a sequence with stronger binding ability (right) through 15 rounds of optimization. Binding affinity was assessed after modifying specific nucleotide positions within the sequence. Representative EMSA gels showed the results of titrating T7 primase with ssDNA templates where the 1st (**b**), 9th (**c**), 10th (**d**), and 11th (**e**) sites being modified to different deoxyribonucleotides, respectively. Gradient titrations with T7 primase to the initial (**f**) and optimized (**g**) ssDNA sequences were performed. The binding curves were nonlinearly fitted, which corresponded to a substantial increase in binding affinity, with the Kd value decreasing from 80.26 μM to 49.62 μM. Original images can be found at Supplementary Materials.

Iterative optimization was performed to investigate the impact of 1–15 nucleotide sites within 3′ flanks of 5′-TGGTC-3′ on the binding affinity of ssDNA to T7 primase (Figure 3a). In the first optimization, the adenine nucleoside (A) from the first site of 3′ flanks of 5′-TGGTC-3′ in sD3 was modified to T, C, or G, respectively, then the binding affinity of modified sD3 to T7 primase was measured by EMSA. For each set of modifications, the difference between the lowest (indicating the strongest binding affinity) and the highest (indicating the lowest binding affinity) proportion of free ssDNA was used as the influence ratio for this site. As shown in Figure 3b, the modification on sD3 affected the binding affinity of ssDNA to T7 primase, the 1st site being A exhibited the highest binding affinity to T7 primase, while being G resulted in the lowest binding affinity. Hence, the 1st site flanking the 3′ of 5′-TGGTC-3′ in sD3 was optimized as A, then the other sites were optimized in the same way.

In total, 15 rounds of iterative optimization were carried out, among which the 1st to 10th rounds of optimization exhibited impact on the binding affinity of sD3 to T7 primase (Figure 3c,d and Figure S2a–g). Notably, modifications on the 9th and 10th sites of deoxyribonucleotides determined to be C virtually annihilated the binding between ssDNA and T7 primase (Figure 3c,d), while the modifications on the deoxyribonucleotides after the 10th site showed almost no effect on the binding between sD3 and T7 primase (Figure 3e and Figure S2h–k).

**Figure 4:**
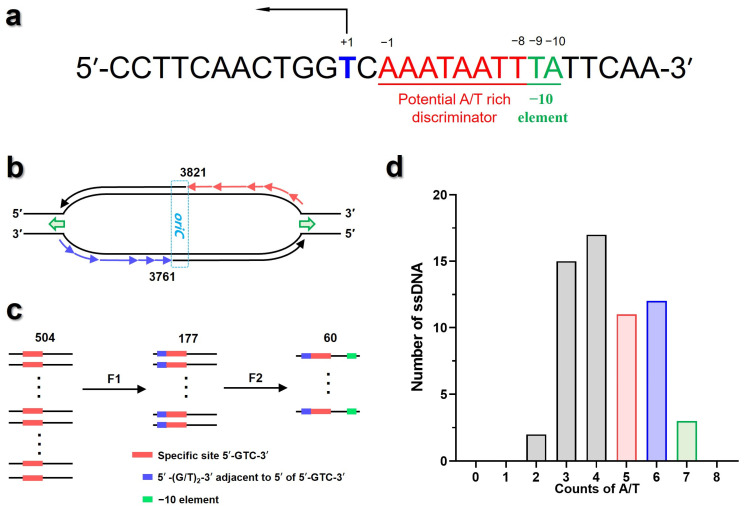
Potential sequence screening through T7 phage genome. (**a**) SIO result of initiation sequence sD3, with the definition of primer synthesis initiation site (blue base), potential A/T-rich discriminator (red region), and −10 element (green region). (**b**) Diagram for DNA regions requiring priming. Discontinuous replication region on negative chain was colored in blue, while that on positive chain was in red. (**c**) Diagram for screening process. A stepwise filtration workflow was shown: 504 occurrences of the trinucleotide 5′-GTC-3′ were observed across the lagging strand, 177 sites containing the extended pentanucleotide 5′-(G/T)_2_GTC-3′ were identified, and 60 candidate sites were defined with specific sequence features within the −10 element. (**d**) Counts of A and T within potential A/T-rich discriminators for the 60 screened sequences (panel **c**). Columns marked in red, blue, and green stand for sequence cluster with 5, 6, and 7 counts of A/T.

After optimization, an optimized ssDNA was acquired, with a calculated Kd value of 49.62 μM, which was significantly lower than that of the initial ssDNA (Figure 3f,g). Overall, the above results indicated that 9–10th sites of 3′ sequence flanking the 5′-TGGTC-3′ site substantially influence the binding affinity of sD3 to T7 primase. Using the optimized ssDNA as the template, we defined the −10 element as comprising these two sites (Figure 4a), and the top four combinations with strong binding affinity were 5′-TA-3′, 5′-TG-3′, 5′-GA-3′, and 5′-GG-3′. Moreover, the templates with the 1st to 8th sites of 3′ sequence flanking the 5′-TGGTC-3′ site being A or T exhibited stronger binding affinity to the primase, this region was defined as A/T-rich discriminator (Figure 4a). These suggested that the sequence context of the flanks adjacent to 3′ end of 5′-TGGTC-3′ site could be an additional feature affecting binding affinity of ssDNA and the T7 primase.

### 3.2. Screening the Potential Initiation Sites from T7 Phage Genome

By combining the previous studies [12,14,21,22] with the results of iterative optimization conducted here, the ssDNA templates preferably being recognized by T7 primase were characterized by the following features: (1) the pentanucleotide 5′-(G/T)_2_GTC-3′ site; (2) the −10 element being 5′-TA-3′, 5′-TG-3′, 5′-GA-3′ or 5′-GG-3′, the top four combinations with strong binding affinity; and (3) A/T-rich discriminator, the total number of A and T exceeds 4 within 1st to 8th sites of 3′ sequence flanking the 5′-(G/T)_2_GTC-3′ site. These features were hence applied to screen the initiation sites in the T7 phage genome.

In T7 phage genome, an A/T-rich cluster within the oriC located from 3761 to 3821 [32]. There was a 5′-GGGTC-3′ site within AT-rich cluster, but this pentanucleotide site was not used for priming [33]. Thus, T7 primase should recognize the specific priming sites within the region of 1–3761 on the negative chain, and that of 3821–36,942 on positive chain (Figure 4b). Within the above regions, 177 pentanucleotide 5′-(G/T)_2_GTC-3′ sites were identified. When designating the −10 element, 60 ssDNA templates stood out (Figure 4c). After that, these ssDNA templates were aggregated based on the potential A/T-rich discriminator (Figure 4d). Accordingly, those with A/T-rich 3′ flanks were screened out as the potential active templates. Overall, 26 ssDNA from T7 genome were selected for functional evaluation (Table 1).

### 3.3. Binding Affinity of Candidate ssDNA to T7 Primase

Among the 26 screened ssDNA sequences, 7 of them were located on the negative chain, and the other 19 sequences on the positive chain. For binding affinity evaluation, 2 negative control sequences, named C1 (5′-CGTGATGC-TGGTC-GAACTGGC-CC-CCTTT-3′) and C2 (5′-CGTGATGC-TGGTC-GAACTGGC-TA-CCTTT-3′), were synthesized. Specifically, the C1 sequence contained the pentanucleotide 5′-TGGTC-3′, with only three A/T counts within the potential A/T-rich discriminator and the cytosines (C) within −10 element. The C2 was modified from C1 sequence by substituting the −10 element with 5′-TA-3′.

**Figure 5:**
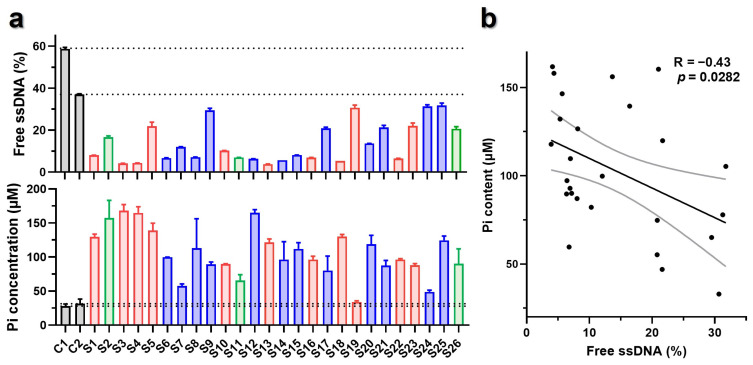
Activity evaluation of the potential 26 sequences. (**a**) Binding affinity of the 26 screened sequences to T7 primase and primer synthesis activity of T7 primase with the 26 ssDNA templates. Columns marked in red, blue, and green stand for sequences with 5, 6, and 7 A/T counts within potential A/T-rich discriminators corresponding to those in Figure 4c; the results from C1 and C2 are colored in gray. (**b**) Correlation analysis between the binding affinity of 26 ssDNA to T7 primase and primer synthesis activity of T7 primase, with the sequences above as templates.

The binding affinity of C1, C2, and 26 potential ssDNA templates were measured by EMSA, with the results indicating 26 potential ssDNA templates had higher affinity to T7 primase than that of C1 and C2 (Figure 5a, top). For validation, six ssDNA templates with different binding affinity to T7 primase were selected to acquire the Kd values. As shown in Figure S3, the Kd values derived from validation further confirmed the outcomes of EMSA.

### 3.4. Primer Synthesis Activity of T7 Primase with Candidate ssDNA as Template

In addition, non-radioactive primase–pyrophosphatase activity assays were performed with the 26 screened ssDNA as templates. As shown in Figure 5a (bottom), the template of C1 had the lowest primer synthesis activity, while C2 had slightly higher primer synthesis activity than C1. Most of the 26 ssDNA templates exhibited significantly higher primer synthesis activity than C1 and C2.

Correlation analysis indicated a positive correlation between binding affinities of ssDNA to T7 primase and primer synthesis activities, suggesting that the stronger binding affinities of ssDNA to T7 primase mostly meant higher primer synthesis activities (*p* < 0.05, Figure 5b). Lipps reported that binding specificity was more relaxed than priming specificity. In primer synthesis, the incorporation of NTP might cause additional steric/chemical constrained to priming specificity of T7 primase [34]. Despite all this, 26 candidate templates still had stronger binding affinity and higher primer synthesis activity, which included potential priming sites for DNA replication.

### 3.5. Mapping the Active Priming Sites onto the Genome of T7 Phage

**Figure 6:**
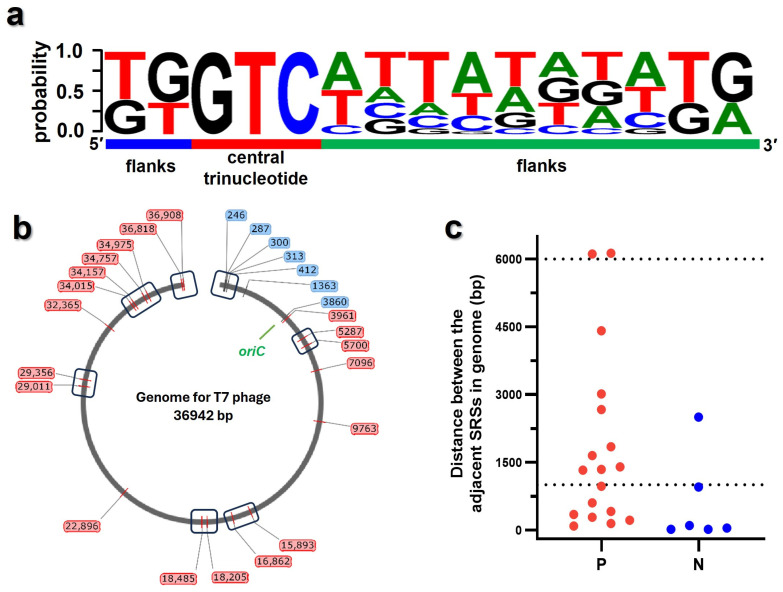
Distributions of the potential priming sites among T7 phage genome. (**a**) Features of the 26 screened sites are as follows: conservation of the core pentanucleotide, variability within the −10 element, and A/T richness in the discriminatory region. (**b**) Locations of the 26 screened sites on the T7 phage genome. Boxes represent seven group of sites with intervals much less than 1000 bp, where each can be regarded as an approximate priming site. (**c**) Distance between the adjacent sites. Positions colored in blue, red, and green stand for priming sites on negative and positive strands, as well as oriC replication origin, respectively. The two dashed lines define the theoretical range of the intervals (1000–6000 bp).

The sequence features of 26 potential priming sites were acquired (Figure 6a), including 2-nt 5′ flanks, central trinucleotide, and 10-nt 3′ flanks, which were consistent with the screening criteria in Section 3.1, and were mapped onto the T7 genome (Figure 6b). These sites were distributed throughout the genome. Lee et al. reported there was no Okazaki fragment with the length below 1000 bp during T7 DNA replication [13], which meant the interval of potential priming sites should be beyond 1000 bp. Here, the distance between two adjacent sites were calculated, with two groups of adjacent sites spaced slightly more than 6000 bp apart. However, some adjacent sites clustered with the intervals much less than 1000 bp (Figure 6c). As a result, 19 sites formed seven clusters (Table S2). In each cluster, only one priming event would occur during Okazaki fragment synthesis, so one cluster can be regarded as one priming site (Figure 6b, box). In total, 14 sites with the intervals ranging from ~1000 bp to ~6000 bp were defined, consistent with the literature reports [13]. Furthermore, sequences reported as the first priming sites on each lagging strand were included as sites S7 (3860) and S8 (3961) [32]. Therefore, we conclude that the 26 originally identified sites (or at least the 14 consolidated sites) might represent the initiation sites recognized by T7 primase during DNA replication.

### 3.6. The ZBD Might Interact with the −10 Element in 3′ Flanks of the Pentanucleotide Site

As described above, the −10 element in 3′ flanks of the priming site played a prominent role in the interaction between ssDNA template and T7 primase, which implied that the −10 element might contribute to the specific ssDNA template recognition by T7 primase. For the primase of *M. tuberculosis*, the ZBD domain was reminiscent of sigma factor of bacterial RNA polymerase and played important roles in ssDNA template recognition, primer synthesis and delivery [35]. For T7 primase, the RPD recognized the pentanucleotide site in ssDNA template, probably, the ZBD might interact with the −10 element. In the RPD of T7 primase, the regions for primer synthesis and delivery were positive-charged [36]. The residues K122, K128, K131 and K137 contributing to RNA synthesis activity all lied within these patches [27]. Coincidently, a positive-charged patch was also observed in the ZBD of T7 primase (Figure S4a), suggesting its important role in primase–template interactions.

**Figure 7:**
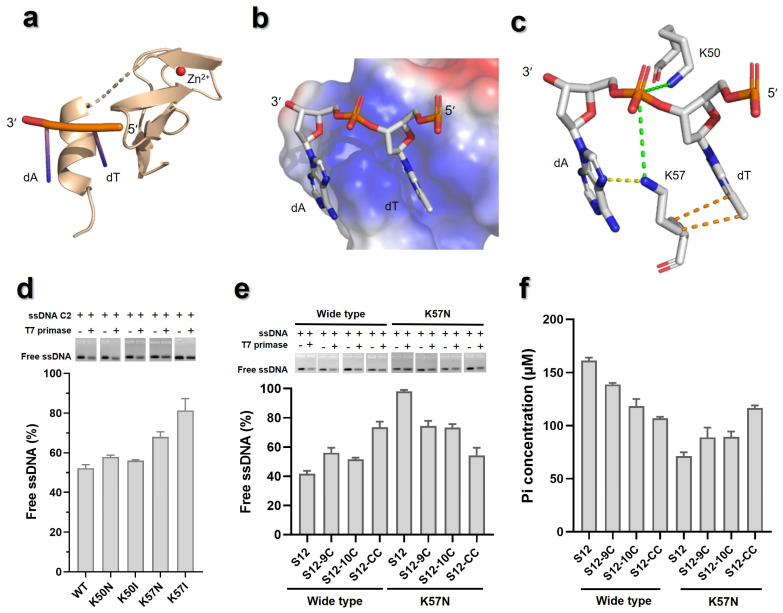
Role of K57 on the interaction between ZBD and −10 element. (**a**) Structure of the ZBD/5′-TA-3′ complex (docked). ZBD is colored in light orange while red sphere represents Zinc ion. (**b**) Surface electrostatics potential of the ZBD/5′-TA-3′ complex (docked). Red areas represent negative-charged surface, while blue areas represent positive-charged surface. (**c**) Key residues K50 and K57 in ZBD which interact with the 5′-TA-3′ dinucleotide were detected in the docked structure. H-bond, salt bridge and hydrophobic interaction were displayed by dashed line and colored in yellow, green and orange, respectively. (**d**) Binding affinity of ssDNA template C2 for T7 primase and mutants. (**e**) Binding affinities of ssDNA templates S12 (with the −10 element being 5′-TA-3′) and its derivatives for T7 primase and mutants. (**f**) Primer synthesis activities of wild type T7 primase compared with K57N mutant using different ssDNAs as templates. Original images can be found at Supplementary Materials.

To test the hypothesis above, T7 primase–ssDNA template docking was conducted using the HDOCK service. Due to the high flexibility of the full-length T7 primase as well as the ssDNA template, docking between domains of T7 primase and recognition sites of ssDNA was performed. Specifically, the oligo of 5′-GGGTC-3′ was docked to the RPD (Figure S4b), and the dinucleotide of 5′-TA-3′, which exhibited the strongest binding affinity within −10 element, was docked to the ZBD (Figure 7a). Nucleotides and Mg^2+^ were provided in order to improve the accuracy of docking.

In the docked structure of RPD/5′-GGGTC-3′ complex, the oligo ssDNA bound to the positive-charged patch on the RPD, another positive-charged patch accommodated nucleotides might be primer binding region (Figure S4c) [37,38]. The RPD/ssDNA complex was well superimposed with the RPD/CTP complex from *Staphylococcus aureus* (PDB: 4EE1) (Figure S4d). The ATP and Mg^2+^ ions in the docked structure matched well with CTP and Mn^2+^ ions in the crystal structure of RPD/CTP complex [39]. In addition, the oligo of 5′-GGGTC-3′ shared the same polarity with ssDNA in the RPD/ssDNA complex of *E. coli* (PDB: 3B39) [40]. In the docked RPD/5′-GGGTC-3′ complex, the residues of D207, D209 and D237 stabilized the two Mg^2+^ ions, hydrogen bonds (H-bond) were formed between K122 and two oxhydryl groups of ribose, while K128 and R84 formed salt bridge and H-bond with phosphate group of ATP, respectively (Figure S4e), consistent with previous reports [27,38,41]. All key residues identified in the docked structure were conserved in DnaG of *E. coli* and *S. aureus* (Table S3) [39]. Besides, H-bonds were formed between ATP and thymine on ssDNA template, as well as the pairing between CMP and guanine (Figure S4e). These results implied the reliability of the docked structure.

In the ZBD/5′-TA-3′ complex, the dinucleotide was docked into a positive-charged patch in the ZBD (Figure 7b). The residue of K50 was observed interacting with phosphate backbone of the dinucleotides. The side chain of K57 stretched into interspace between the two nucleotides, forming salt bridged with phosphate backbone of 5′-TA-3′. In addition, K57 employed H-bond and hydrophobic interactions with the bases of dA and dT, respectively (Figure 7c). To clarify the role of K57 in template selection, the dinucleotides 5′-CC-3′, which exhibited the weakest binding affinity to T7 primase during SIO process, was docked into the ZBD. The structure of ZBD/5′-CC-3′ demonstrated an unfavorable pair, since 5′-CC-3′ deviated from the position where 5′-TA-3′ was located. Thus, K50 and K57 were observed only interacting with the phosphate backbone (Figure S5). This was consistent with the affinity evaluation data described in Section 3.3. Specifically, it was reported that lysine had low affinity to cytosine versus guanine, while asparagine and isoleucine had low affinity to guanine versus cytosine [42]. To test the importance of residues K50 and K57, the mutants of K50N, K50I, K57N, and K57I were prepared. C2 ssDNA with optimal binding base composition (5′-TA-3′ in its −10 element; Section 3.3) was employed for binding assay. As shown in Figure 7d, K57N and K57I showed lower C2 ssDNA binding capacity (about 15–30%) than the WT, while K50N and K50I showed binding affinity at the WT level. These results implied K57 might play crucial role in primase–ssDNA interaction.

To further investigate the role of K57, the S12 ssDNA in Table 1 with 5′-GG-3′ in its −10 element was selected as template, where modifications in the −10 element generated the ssDNA of S12-9C, S12-10C, and S12-CC (Table S4). The effect of guanine-to-cytosine substitution in ssDNA on the interaction between ssDNA and T7 primase was investigated. The binding affinity and primer synthesis activity of these four templates were measured. As shown in Figure 7e, the binding affinity of ssDNA to T7 primase decreased when either G of −10 element was replaced by C, while the double replacement greatly weakened the binding of such ssDNA to T7 primase. In contrast, the binding affinity to K57N increased for ssDNA of S12-9C and S12-10C, while the ssDNA of S12-CC showed much stronger binding affinity to T7 primase.

For T7 primase, the primer synthesis activity decreased when the either G of −10 element was replaced by C. The double replacement caused a drastic decrease in activity (about 30% activity of WT). In contrast, the primer synthesis activity of K57N increased when either G was replaced by C, where K57N had a much higher efficiency for primer synthesis with S12-CC than that with S12-9C or S12-10C (Figure 7f). Taken together, these results implied that K57 might interact with −10 element, thus assisting the ZBD in recognizing the ssDNA template, which was consistent with the docking results.

## 4. Discussion

In the T7 phage, the primase initiates primer synthesis from the specific pentanucleotide 5′-(G/T)_2_GTC-3′ [19,20], with a total of 177 pentanucleotide sites identified in the lagging strand region. The length of the Okazaki fragment in the T7 phage is reported to range from 1000 to 6000 bp [13], implying that only ~12 sites are required for active priming. In this work, the preferred features of 3′ 10-nt flanks of pentanucleotide site were addressed based on the Sequence Iterative Optimization Strategy. It was found that the 3′ flanks of pentanucleotide sites, including a −10 element and the potential A/T-rich discriminator, contribute to the binding specificity between T7 primase and ssDNA, which resulted in the discovery of potential 26 sites among the genome. These sites may serve as priming sites for T7 genome replication, despite that an unknown mechanism may still exist for selecting one site from those neighbors. In addition, K57 of the ZBD was investigated as a key residue responsible for interacting with −10 element, thus supporting the selectivity of T7 primase.

Afek et al. reported that T/G rich in both 5′ and 3′ flanks of 5′-GTC-3′ increased affinity of T7 primase for template [12,14]. However, in bacteria and bacteriophage, DnaB helicase and DnaG primase constitute the core part of the replisome [27,43]. During DNA replication in vivo, the replisome slides on the lagging strand from 5′ to 3′, where the newly exposed 5′ ssDNA sequence will be bound by single-strand DNA binding proteins rapidly [44,45]. Asymmetrically, it seemed that the 3′ flanking sequence of 5′-(G/T)_2_GTC-3′ should contribute much more to the T7 primase binding, which was consistent with a previous report that an additional sequence flanking the 5′ of pentanucleotide 5′-(G/T)_2_GTC-3′ had no effect on the T7 primase–ssDNA interaction [21]. Our data showed that both the pentanucleotide 5′-(G/T)_2_GTC-3′ and 3′ flanking sequence contribute to priming-site selection, which might suggest a readout mechanism of multiple-site recognition for T7 primase. Of note, multiple-site recognition was also observed in T7 RNA polymerase and *E. coli* RNA polymerase [37,46,47,48]. For T7 primase, except for the central pentanucleotide, we found the −10 element and the potential A/T-rich discriminator contributed to SRS recognition. Among them, the −10 element might be recognized by the ZBD, like the sigma factor of RNA polymerase in *E. coli*. By multiple-site recognition, the T7 primase may initiate priming by choosing the active ones among the genome, which facilitates the phage in accomplishing genomic DNA duplication in efficient way. However, the fact that the T7 primase is able to synthesize various dinucleotides at a low rate in the absence of a template demonstrated that the stringency of T7 primase is relatively low [6]. Thus, it should be noted that the −10 element and A/T-rich features were at least partially involved in priming site selection. The lack of these two features might lead to decrease in affinity and pre-matured priming. DNA replication is a highly orchestrated process that must be completed with remarkable speed and fidelity. Given the precise spatiotemporal regulation on DNA replication, a selective mechanism for primer synthesis is not only more plausible but also easy to coordinate with successive steps in vivo.

**Figure 8:**
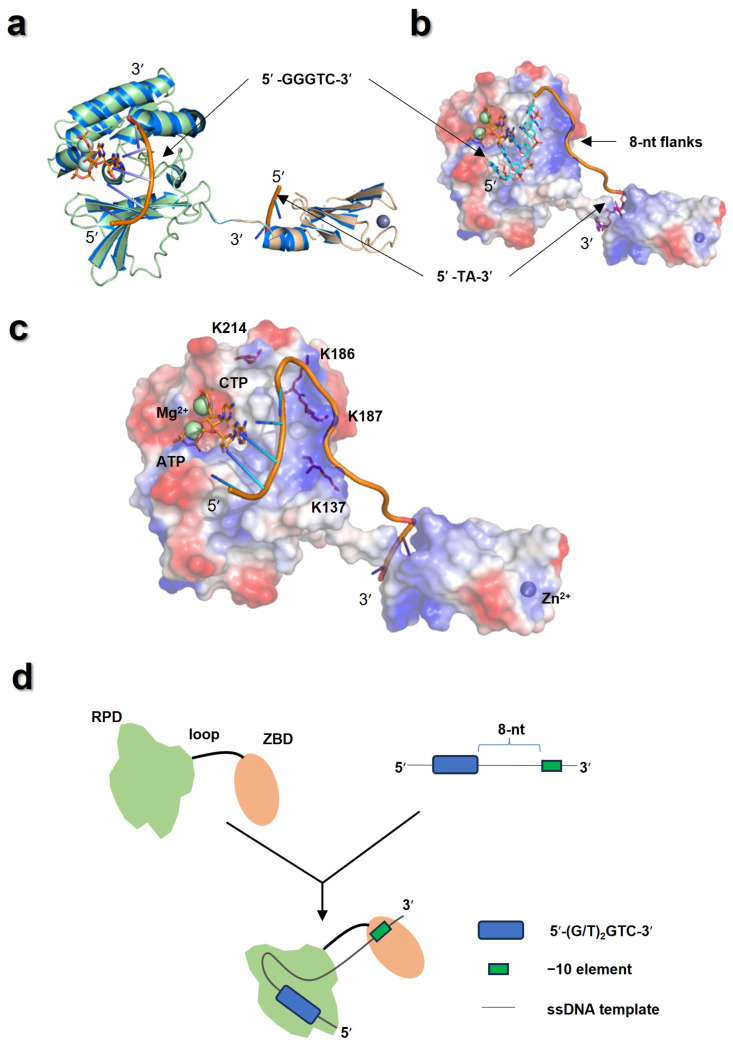
Model for T7 primase recognizing active priming site. (**a**) Superimposition of the RPD/5′-GGGTC-3′ complex (pale green) and the ZBD/5′-TA-3′ complex (light orange) with crystal structure of T7 primase (marine). (**b**) The 8-nt nucleotides oligo (represent of potential A/T-rich discriminator) was aligned along the positive-charged patch; this oligo connected the 5′-GGGTC-3′ sequence and 5′-TA-3′ dinucleotides. Red areas represent negative-charged surface, while blue areas represent positive-charged surface. (**c**) 15-nt ssDNA binding to the T7 primase; the residues of K137, K186, K187, and K214 were located in the nucleic acid-contact regions. Green and black spheres represent Magnesium ion and Zinc ion, respectively. (**d**) Multiple-site recognition model for T7 primase to select priming sites among the ssDNA template.

Previous studies suggested that the ZBD of T7 primase interacted with cryptic cytosine of 5′-GTC-3′ through residues D31 and H33 [6,49,50]. For recognition of 5′-GTC-3′, Lee et al. proposed a model that ATP and CTP firstly bound to NTP pockets in the RPD, which, together with the ZBD, employed multiple interactions to differentiate ssDNA sequences, followed by efficient condensation of NTPs [16]. This model provided a reasonable explanation for how T7 primase tightly bound to specific trinucleotides and initiated primer synthesis. However, how T7 primase selects active priming sites from a large number of candidates before tightly binding remains unknown. In this work, K57 in the ZBD was identified as the residue participating in recognition of -10 element, which suggested that T7 primase selected active priming sites by multiple-site recognition mode, namely the RPD/5′-GGGTC-3′ and ZBD/5′-TA-3′ recognition (Figure 8a). Moreover, the sequence between 5′-GTC-3′ and the −10 element also modulated priming site selection, despite its modest impact on affinity (Section 3.1). The location of 8-nt potential A/T-rich discriminator was sought to fill along the positive-charged patch on the RPD (Figure 8b). In fact, previous studies have identified several regions of the RPD (T132-K144, Y173-K187 and K214-K217) making directly contact with nucleic acids [38]. Among these regions, K186 and K187 form a positively charged surface, which facilitates template binding [51]. K137 has been reported to contribute to primer synthesis without directly involvement in the formation of phosphodiester bonds [27,41]. In DnaG from *S. aureus*, the residues K242 and K321, which are the counterparts of K137 and K214 in T7 primase, respectively (Figure 8c), have been shown to be essential for template binding [39]. In combination with the data above, a model for the T7 primase–ssDNA complex was proposed, where the RPD of T7 primase recognized the 5′-(G/T)_2_GTC-3′ site and potential A/T-rich discriminator, with −10 element being recognized by ZBD (Figure 8d).

Of note, Kato et al. proposed two different conformations of T7 primase. In apo state, the RPD and the ZBD remain separated (open state). After the primase bound to ssDNA and nucleotides, the ZBD becomes associated with the RPD, thus securing template in cis within the active site of the RPD (closed state) [27]. This conformational change allowed residues D31 and H33 to approach 5′-GTC-3′ and contacted with the cytosine, followed by primer synthesis initiation [49]. Similar conformational transitions during recognition and priming were also observed in bacterial RNA polymerase [52]. The model we proposed here might reflect the open state for screening of active priming sites before primer synthesis initiation. In this state, the specific upstream and downstream sequences were recognized by different domains of T7 primase. Once an active template was screened out, the T7 primase carried out conformational change and the ZBD moved toward the RPD to secure the template ssDNA in closed state and initiated primer synthesis [16], followed by primer delivery, which might be determined by the electron microscopy structure of T4 primosome and T7 replisome [53,54]. Further investigations are requested to determine the crystal structure of T7 primase complexed with template and NTP, which can reveal the chemical basis for priming-site selection.

## 5. Conclusions

In conclusion, by using Sequence Iterative Optimization Strategy, we investigated features of 3′ flanks of central pentanucleotide recognized by T7 primase and screened out the potential priming sites for T7 genome replication. Furthermore, K57 of ZBD was found to be involved in protein–ssDNA interactions. This finding provides structural insights into priming site selection. Our findings not only elucidate a mechanism of primer synthesis initiation that may be conserved in T7 and bacterial primases, but they also deliver an efficient methodological platform for probing primase–ssDNA interactions. This study thereby identifies the primase–template interface as a druggable target and paves the way for the development of DnaG-targeting antibiotics.

## Figures and Tables

**Table 1:** 26 ssDNA sequences containing potential priming sites selected from T7 genome.

No.	Chain	Central T	Sequence (5′ to 3′)	Regions
S1	Negative	246	GACTTGATGGGTCTTTAGGTGTAGGCTT	230–257
S2	Negative	287	TTTAGGTCTGGTCTTTATGTTTAAACTT	271–298
S3	Negative	300	TTTAGGTCTGGTCTTTAGGTCTGGTCTT	284–311
S4	Negative	313	TTTATGTAGTGTCTTTAGGTCTGGTCTT	297–324
S5	Negative	412	TCTTTAAGTTGTCTCTCCTTATAGTGAG	396–423
S6	Negative	1363	AGCCAGAGTGGTCTTAATGGTGATGTAC	1347–1374
S7	Negative	3860	AGAACGTTTGGTCATCTTTTCGAAGTTA	3844–3871
S8	Positive	3961	AGCTGGGAGGGTCAGTAAGATGGGACGT	3950–3977
S9	Positive	5287	GGCTGGGCGTGTCAAATTAGCTACATGG	5276–5303
S10	Positive	5700	ATGCCAGATGGTCACGCTTAATACGACT	5689–5716
S11	Positive	7096	CCCTCGTGGTGTCTATAAAGTTGACCTG	7085–7112
S12	Positive	9763	GCAAACGAGTGTCACCTAAATGGTCACG	9752–9779
S13	Positive	15,893	GCGTATATTGGTCTGGATCTTTGTGTTC	15,882–15,909
S14	Positive	16,862	AACGTCCGTTGTCATTAATCCTGAGGCA	16,851–16,878
S15	Positive	18,205	TGTACCGATTGTCTTCTTATGTGGTCCA	18,194–18,221
S16	Positive	18,485	GATTCGGATGGTCAGACTAGATGGTGAA	18,474–18,501
S17	Positive	22,896	GCACCTAGTGGTCAACAGATTGACTCCT	22,885–22,912
S18	Positive	29,011	TATGGTCGGTGTCACTGGTAAGGGCTTT	29,000–29,027
S19	Positive	29,356	ACATAATGGTGTCCCTTATGAGGACTTA	29,345–29,372
S20	Positive	32,365	TACTATCGGTGTCAATAACGATGGTCAC	32,354–32,381
S21	Positive	34,015	ACAGATAGTGGTCTTTATGGATGTCATT	34,004–34,031
S22	Positive	34,157	GGCATCTAGGGTCAGACTCAATGGACGC	34,146–34,173
S23	Positive	34,757	TCGTTGTGTGGTCCTTATGGAGAGACCC	34,746–34,773
S24	Positive	34,975	GGTATCACTGGTCAGTTAACTGGTAGCC	34,964–34,991
S25	Positive	36,818	GCCTCTAATGGTCTATCCTAAGGTCTAT	36,807–36,834
S26	Positive	36,908	TTCCTATAGGGTCCTTTAAAATATACCA	36,897–36,924

The pentanucleotide 5′-(G/T)_2_GTC-3′ sites were underlined.

## Data Availability

The data presented in this study are available on reasonable request from the corresponding author.

## Supplementary Materials

The following supporting information can be downloaded at https://www.mdpi.com/article/10.3390/biom16010078/s1. Table S1: Origin sequences with potential optimization sites; Table S2: Seven clusters of 19 potential priming sites; Table S3: Key residues identified in the docked structure and counterparts in bacterial DnaG; Table S4: Sequences of ssDNA templates used in mutagenesis analysis. Figure S1: Binding affinity of four ssDNA templates to T7 primase; Figure S2: Sequence iterative optimization of sequence flanking the 3′ of 5′-(G/T)_2_-GTC-3′; Figure S3: Gradient titration of ssDNA with T7 primase; Figure S4: Docking between RBD of T7 primase and recognition sites of ssDNA and interaction analysis; Figure S5: Docked structure of ZBD/5′-CC-3′ and potential interaction. File S1: Original Images for Blots.
