# Supplementary material for: Mechanism of T7 Primase Selecting Active Priming Sites Among Genome
Source: Biomolecules. 2026 Jan 3;16(1):78. doi: 10.3390/biom16010078 (PMC12839067; doi:10.3390/biom16010078)

Origin images for Gels

Origin gel image for Figure 3b

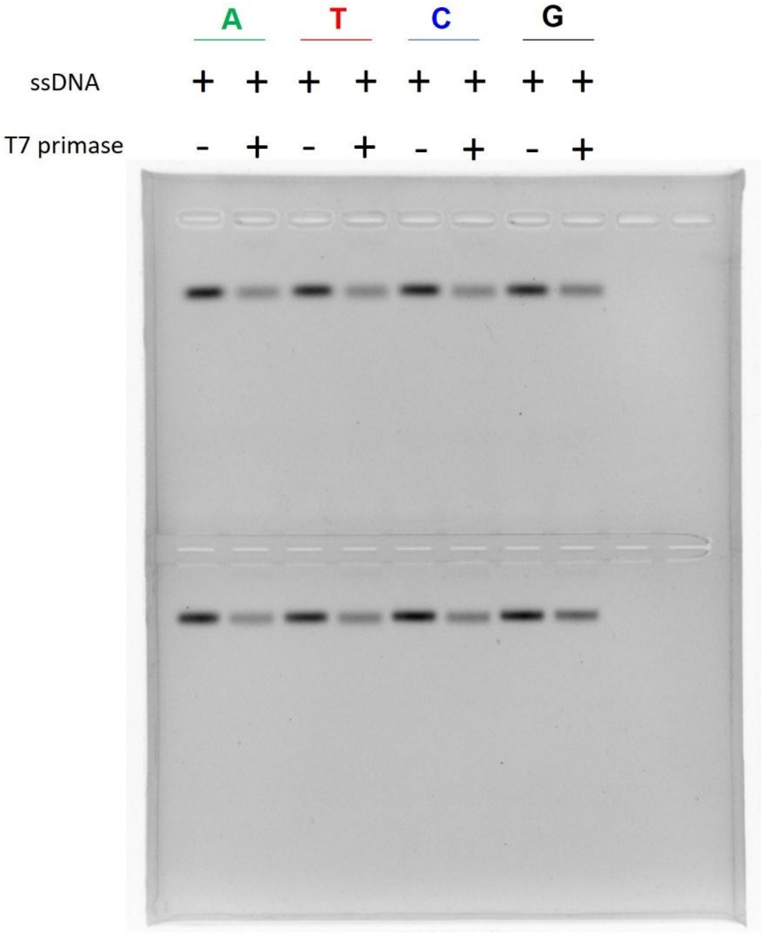

Origin gel image for Figure 3c

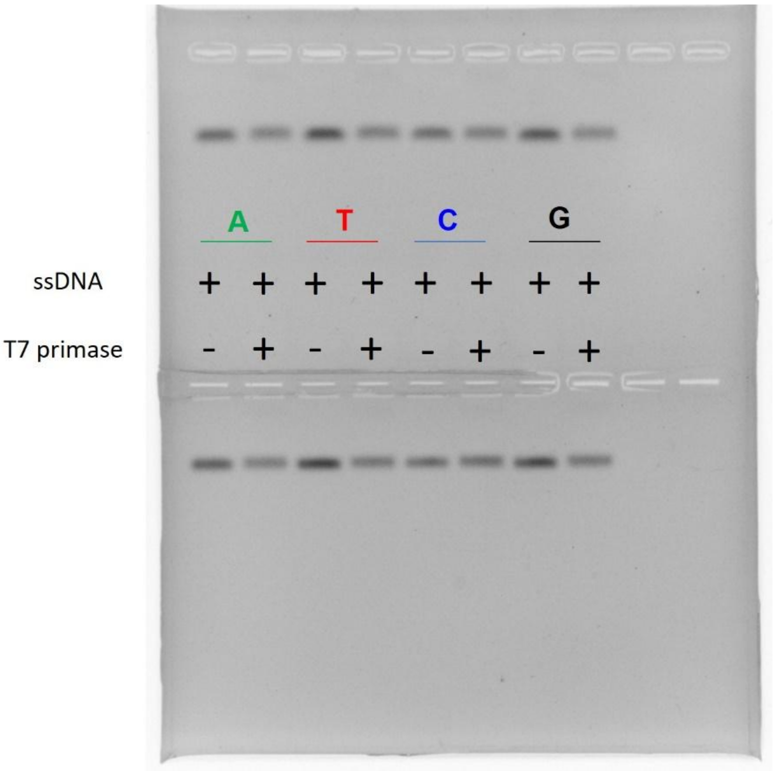

Origin gel image for Figure 3d

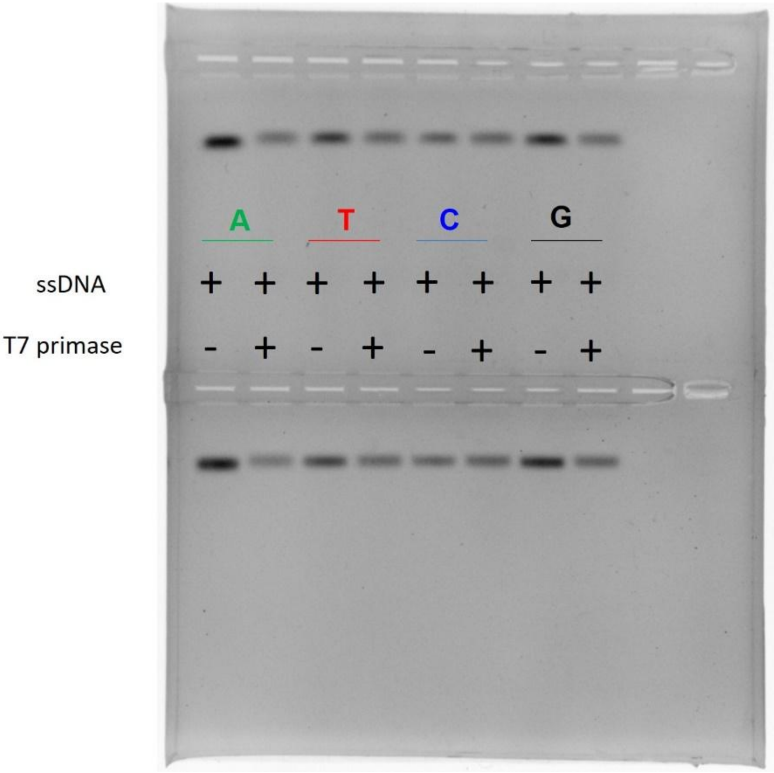

Origin gel image for Figure 3e

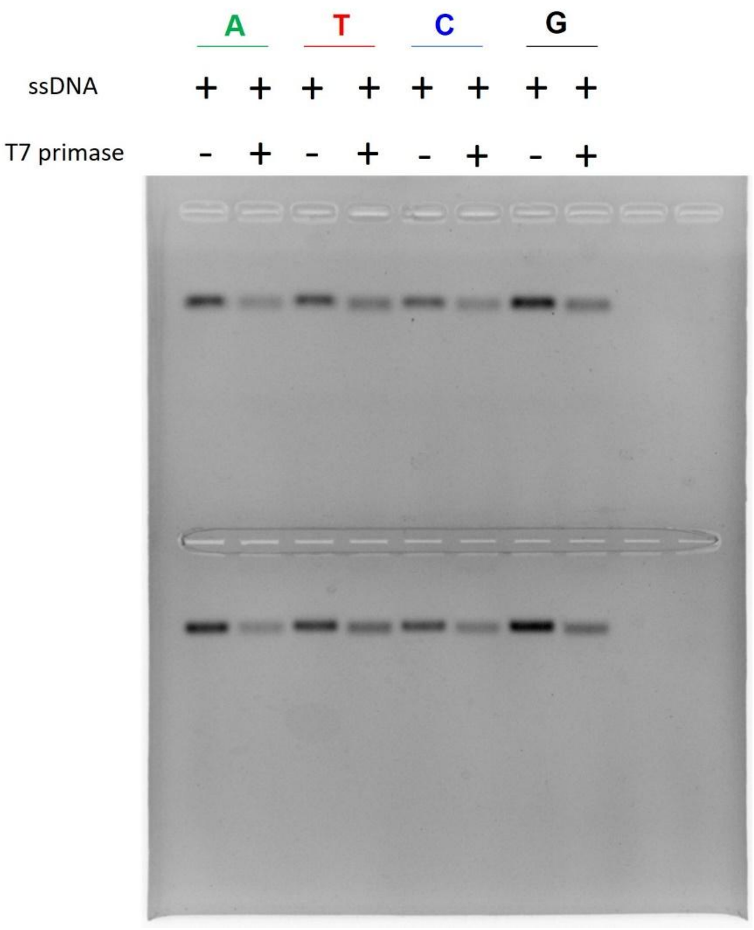

Origin gel image for Figure 3f

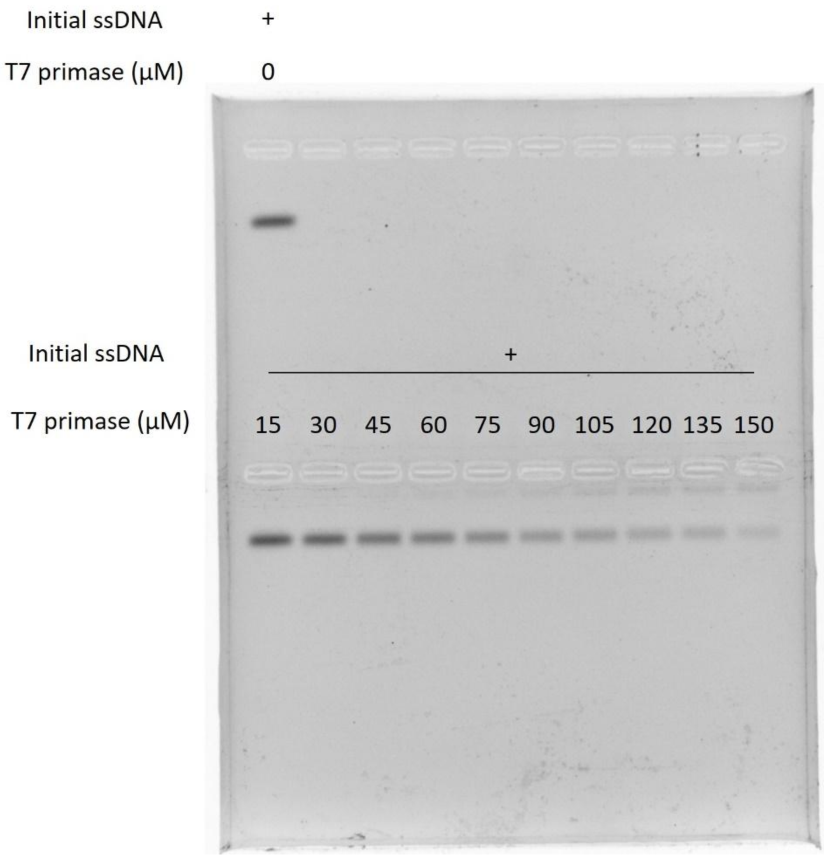

Origin gel image for Figure 3g

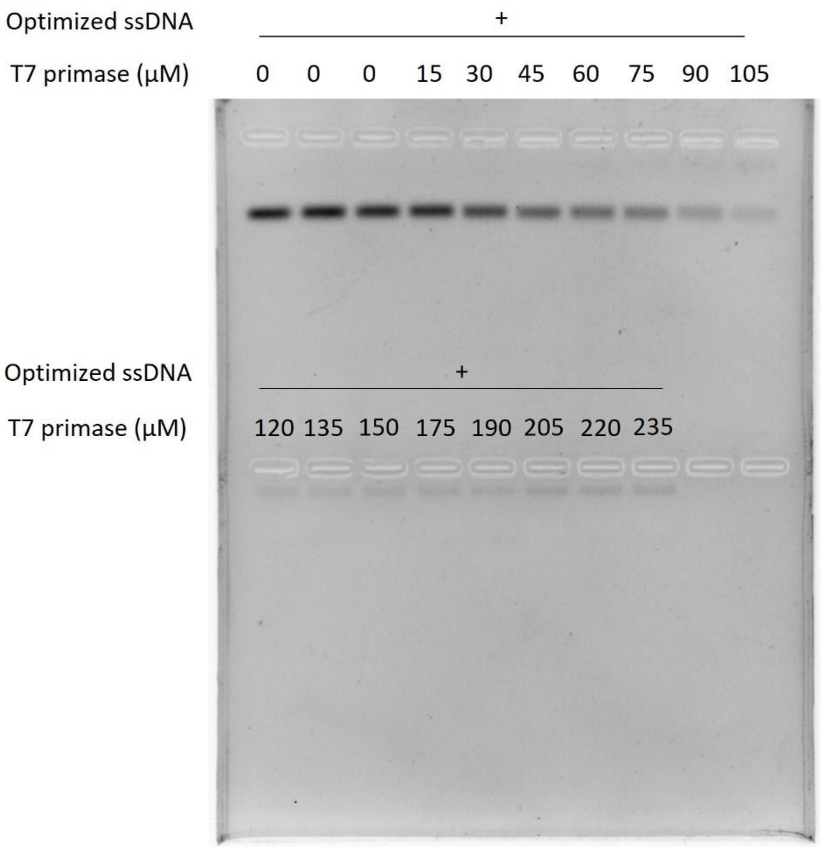

Origin gel image for Figure 7d (WT and mutant K50I, K50N, K57N)

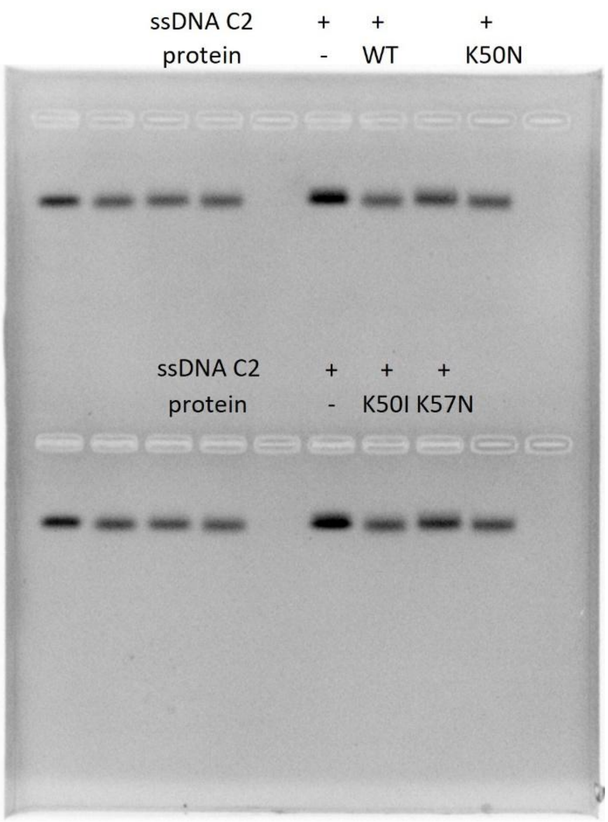

Origin gel image for Figure 7d (mutant K57I)

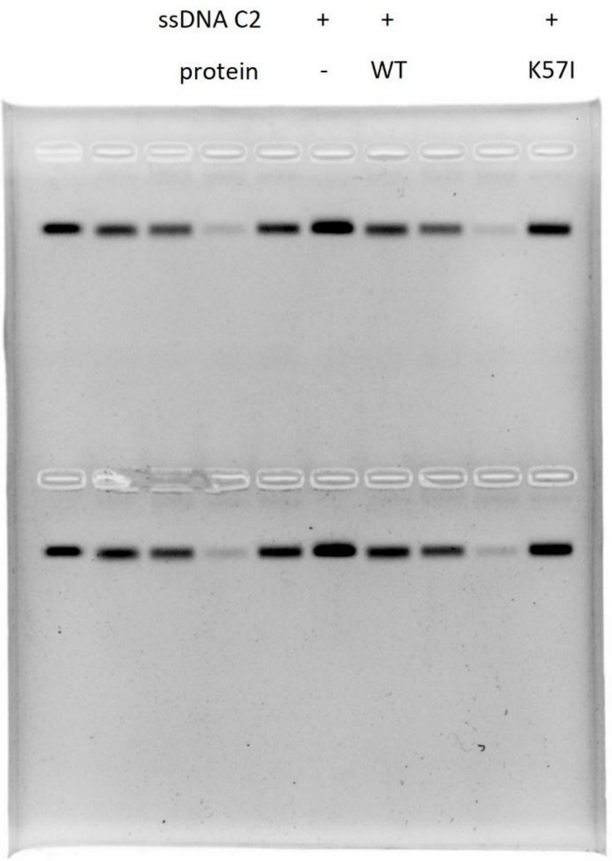

Origin gel image for Figure 7e (WT)

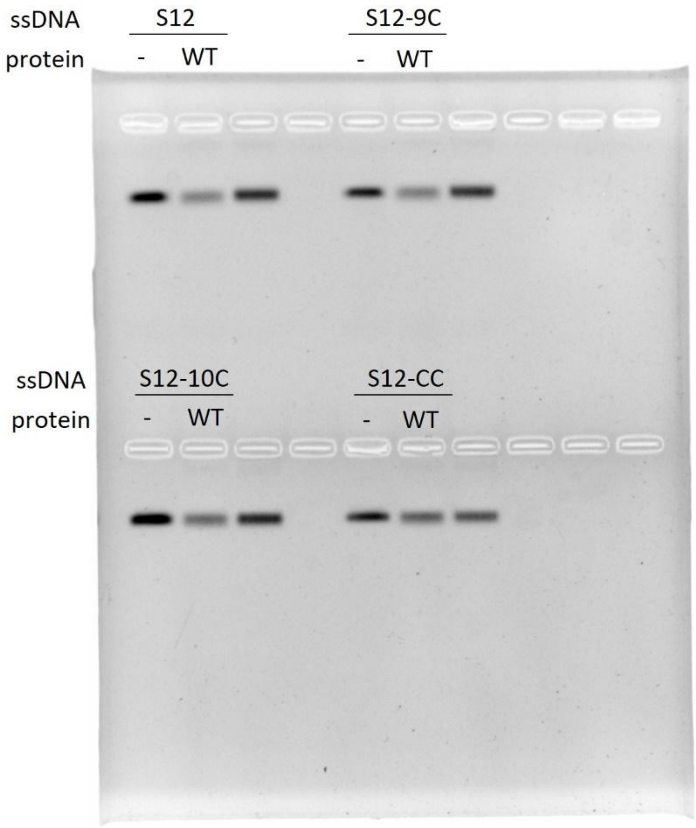

Origin gel image for Figure 7e (WT)

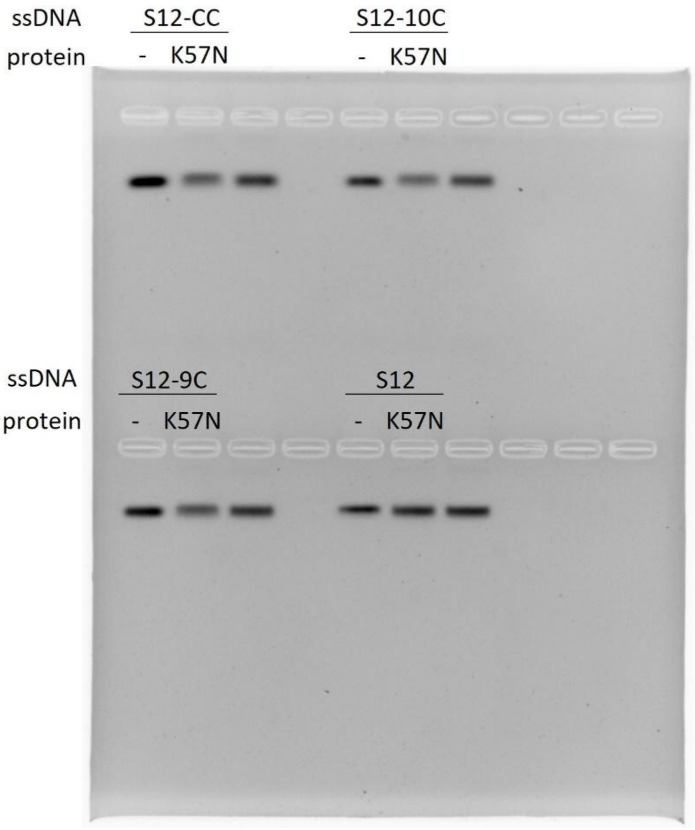

Origin gel image for Figure S1

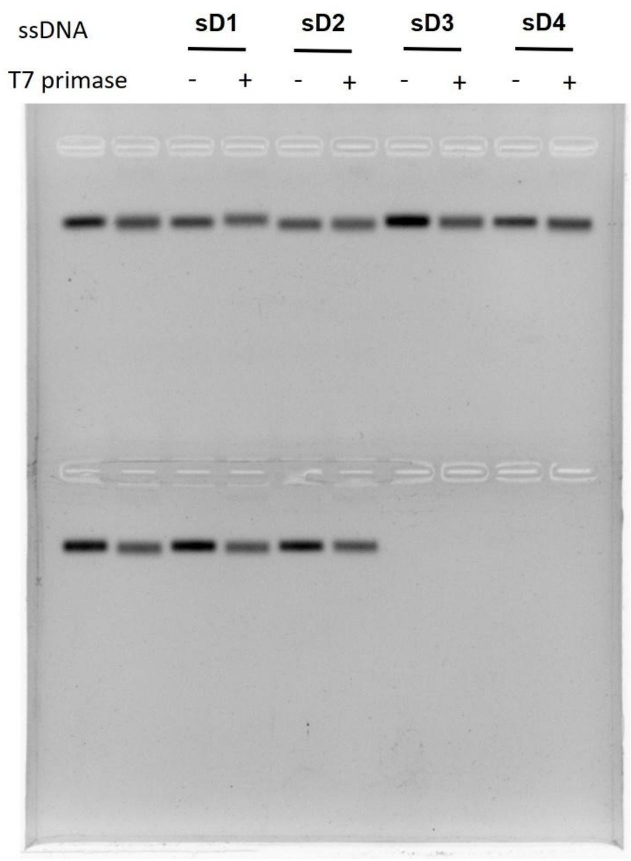

Supplement: Supplementary file 1 [file biomolecules-16-00078-s001.zip › biomolecules-4013430-File S1.pdf]
