# Supplementary material for: Mechanism of T7 Primase Selecting Active Priming Sites Among Genome
Source: Biomolecules. 2026 Jan 3;16(1):78. doi: 10.3390/biom16010078 (PMC12839067; doi:10.3390/biom16010078)
Supplement: Supplementary file 1 [file biomolecules-16-00078-s001.zip › biomolecules-4013430-supplementary (1)-done.pdf]

Table S1. Origin sequences with potential optimization sites.

No.	Sequence (5′ to 3′)
sD1	ACACTTAAGGGTCTTAAAGTTAAACCTT
sD2	AGAACGTTTGGTCATCTTTTCGAAGTTA
sD3	CCTTCAACTGGTCATACATATGGTTCAA
sD4	AGACTTAGCGGTCATTTATGATGACTGG

For sD1-sD3, the pentanucleotide 5′-(G/T)$_2$GTC-3′ sites were underlined. For sD4, the pentanucleotide 5′-CGGTC-3′ was considered unable to interact with T7 primase, thus was used as negative control.

Table S2. Seven clusters of 19 potential priming sites.

No.	Sequence (5′ to 3′)	Regions	Cluster
S1	GACTTGATGGGTCTTTAGGTGTAGGCTT	230-257	
S2	TTTAGGTCTGGTCTTTATGTTTAAACTT	271-298	
S3	TTTAGGTCTGGTCTTTAGGTCTGGTCTT	284-311	I
S4	TTTATGTAGTGTCTTTAGGTCTGGTCTT	297-324	
S5	TCTTTAAGTTGTCTCTCCTTATAGTGAG	396-423	
S9	GGCTGGGCGTGTCAAATTAGCTACATGG	5,276-5,303	II
S10	ATGCCAGATGGTCACGCTTAATACGACT	5,689-5,716	
S13	GCGTATATTGGTCTGGATCTTTGTGTTC	15,882-15,909	III
S14	AACGTCCGTTGTCATTAATCCTGAGGCA	16,851-16,878	
S15	TGTACCGATTGTCTTCTTATGTGGTCCA	18,194-18,221	IV
S16	GATTCGGATGGTCAGACTAGATGGTGAA	18,474-18,501	
S18	TATGGTCGGTGTCACTGGTAAGGGCTTT	29,000-29,027	V
S19	ACATAATGGTGTCCCTTATGAGGACTTA	29,345-29,372	
S21	ACAGATAGTGGTCTTTATGGATGTCATT	34,004-34,031	
S22	GGCATCTAGGGTCAGACTCAATGGACGC	34,146-34,173	VI
S23	TCGTTGTGTGGTCCTTATGGAGAGACCC	34,746-34,773	
S24	GGTATCACTGGTCAGTTAACTGGTAGCC	34,964-34,991	
S25	GCCTCTAATGGTCTATCCTAAGGTCTAT	36,807-36,834	VII
S26	TTCCTATAGGGTCCTTTAAAATATACCA	36,897-36,924	

Table S3. Key residues identified in the docked structure and counterparts in bacterial DnaG.

Key Residue in docked structure	Conserved residue in *E. coli*	Conserved residue in *S. aureus*
R84	R146	R146
K122	N232	N233
K128	K229	K230
D207	D309	D310
D209	D311	D312
D237	D345	D343

Table S4. Sequences of ssDNA templates used in mutagenesis analysis.

No.	Sequence (5′ to 3′)
C2	CGTGATGCTGGTCGAACTGGCTACCTTT

S12	GCAAACGAGTGTCACCTAAATGGTCACG
S12-9C	GCAAACGAGTGTCACCTAAAT*C*GTCACG
S12-10C	GCAAACGAGTGTCACCTAAATG*C*TCACG
S12-CC	GCAAACGAGTGTCACCTAAAT*CC*TCACG

The pentanucleotide 5′-(G/T)$_2$GTC-3′ sites were underlined.

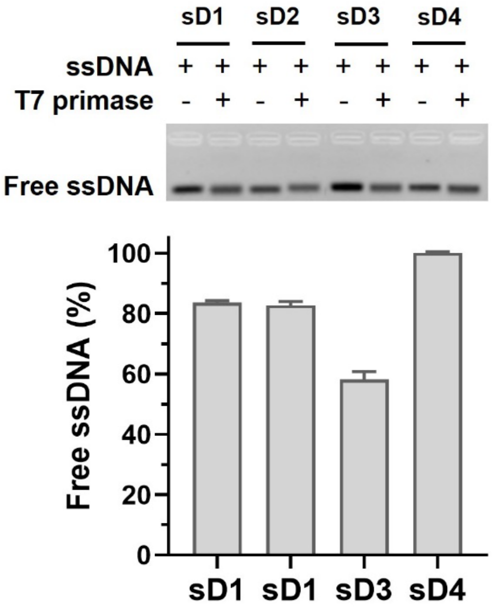

Figure S1. Binding affinity of four ssDNA templates to T7 primase. The ssDNA of sD3 was used for Sequence Iterative Optimization, and the ssDNA of sD4 as negative control.

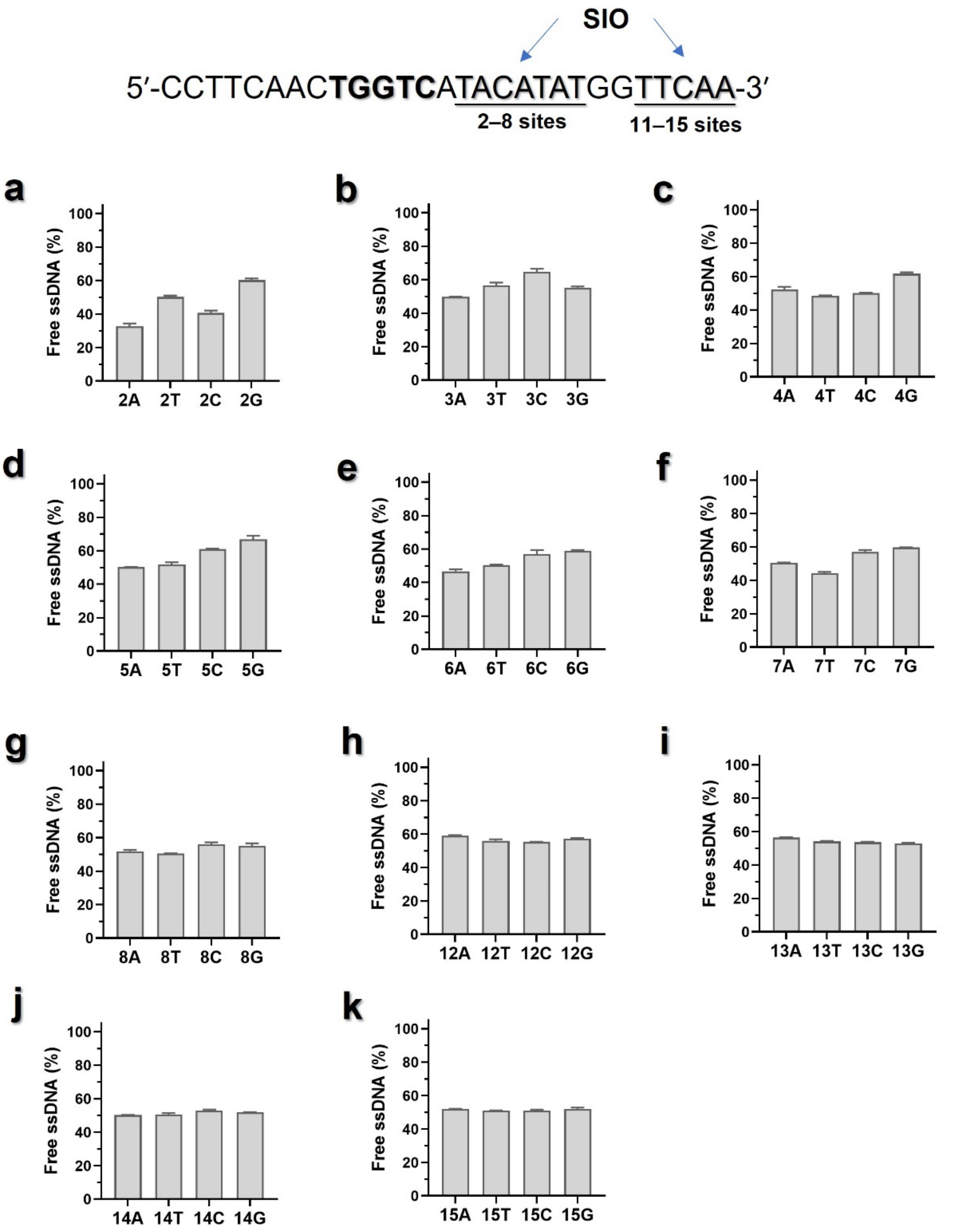

Figure S2. Sequence iterative optimization of sequence flanking the 3′ of 5′-(G/T)2-GTC-3′. (a)-(g), ssDNA with modifications on 2nd to 8th sites in 3′ flank of 5′-(G/T)$_2$-GTC-3′. (h)-(k), ssDNA with modifications on 12th to 15th sites in 3′ flank of 5′-(G/T)$_2$-GTC-3′.

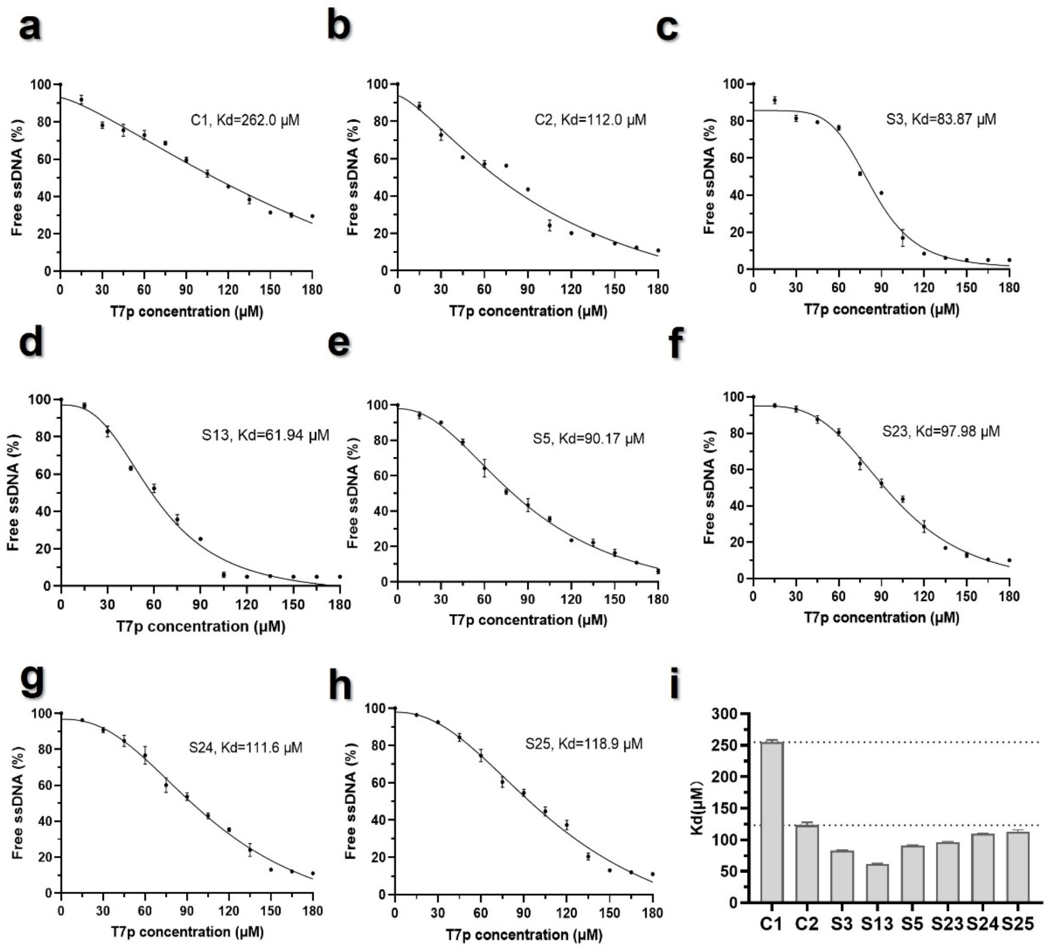

Figure S3. Gradient titration of ssDNA with T7 primase. Analysis on the binding affinity of T7 primase to different ssDNA templates and acquisition of the Kd value, respectively.

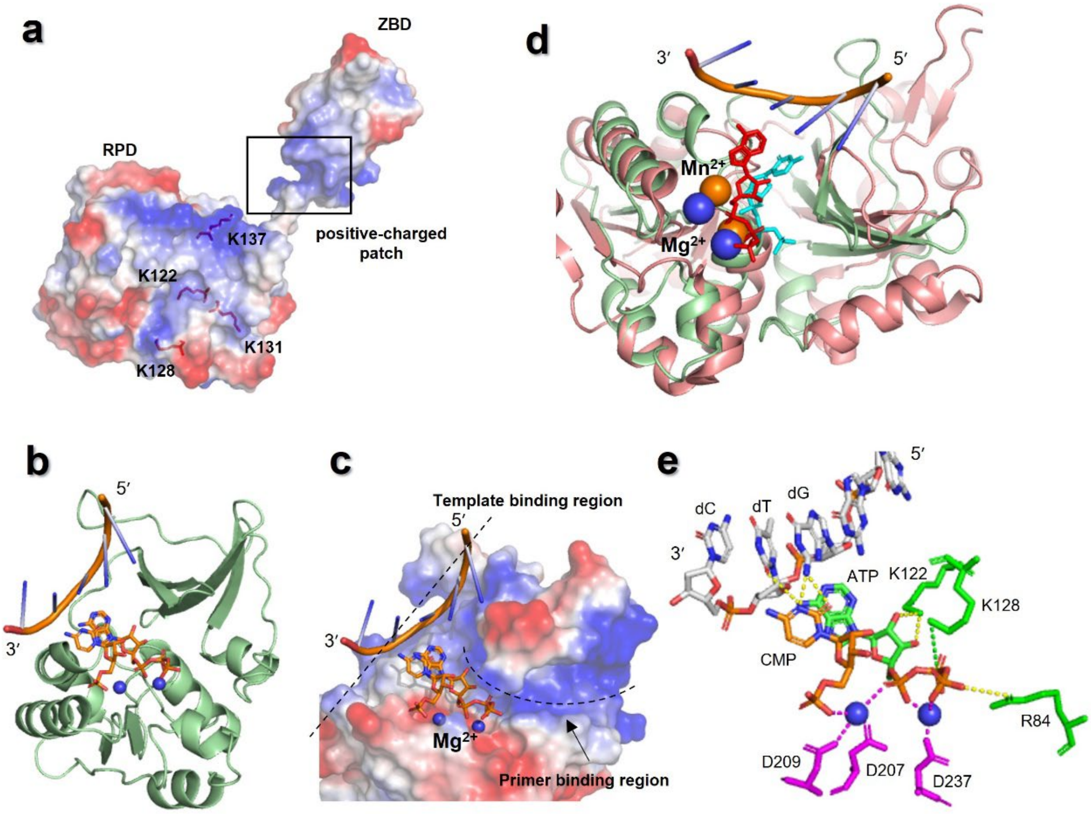

Figure S4. Docking between RBD of T7 primase and recognition sites of ssDNA and interaction analysis. (a) Surface electrostatics potential of T7 primase. Four key residues were labeled. (b) Structure of the RPD/5′-GGGTC-3′ complex (docked). RPD domain is colored in pale green. (c) Surface electrostatics potential of the RPD/5′-GGGTC-3′ complex (docked). (d) Superimposition between the RPD/5′-GGGTC-3′ complex (docked) (colored in pale green) and the RPD/CTP complex of *S. aureus* DnaG (colored in pink). ATP, Mg^{2+} from the docked structure and CTP, Mn^{2+} from DnaG were colored in red, blue, cyan and orange, respectively. (e) Interactions between key residues and Mg^{2+} (blue sphere) and nucleotides (orange sticks). H-bonds are formed between A-T and C-G pairs. Residues interacting with metal ion and nucleotides were colored in purple and green, respectively.

https://doi.org/10.3390/biom16010078

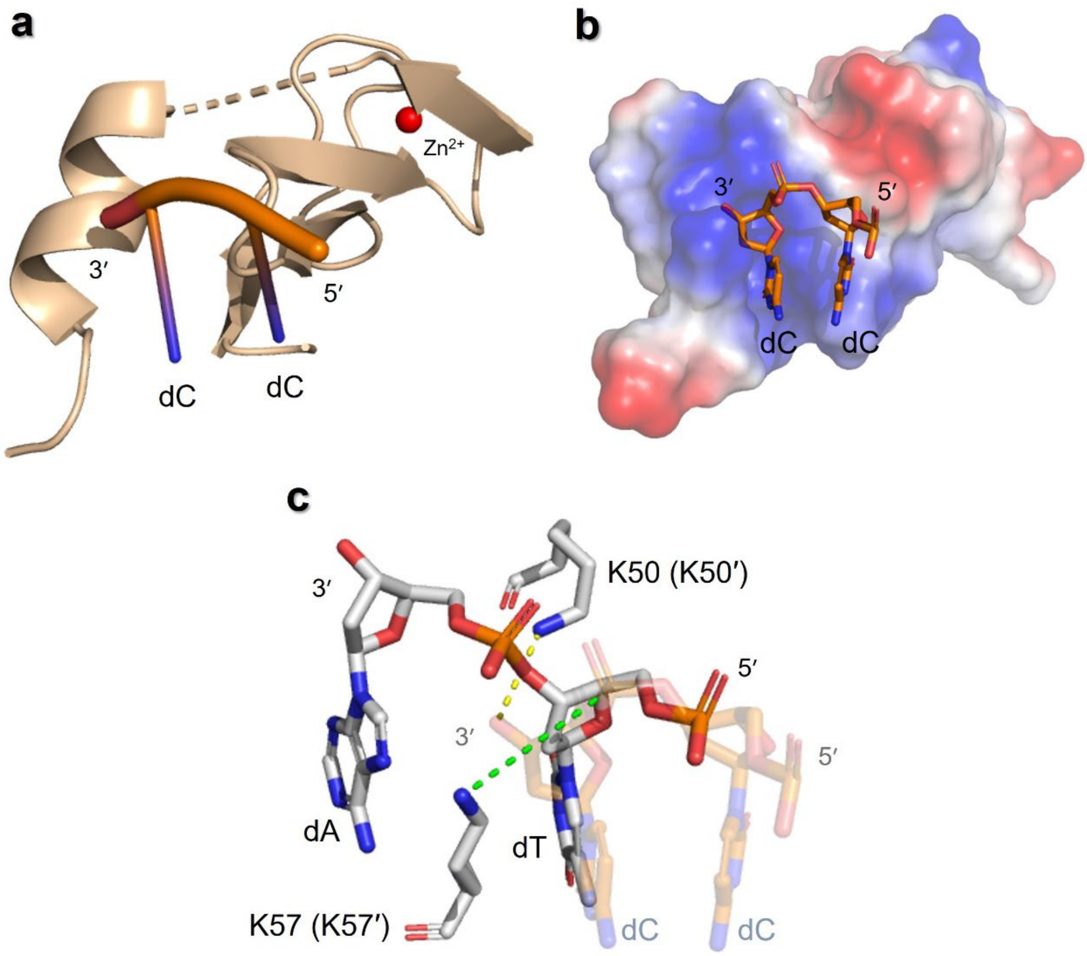

Figure S5. Docked structure of ZBD/5′-CC-3′ and potential interaction. (a) Scheme 5. CC-3′ complex (docked). The ZBD is colored in light orange while red sphere represents for Zinc ion. (b) Surface electrostatics potential of the ZBD/5′-CC-3′ complex (docked). Red areas represent negative-charged surface, while blue areas represent positive-charged surface. (c) Position of 5′-TA-3′ dinucleotide versus 5′-CC-3′ dinucleotide (transparency sticks) in the docked structures. H-bond, and salt bridge were displayed by dashed line and colored in yellow and green, respectively.
